# Hospital operative volume impacts surgical outcomes for patients with T4 rectal cancer following neoadjuvant chemoradiation: a national cancer database analysis

**DOI:** 10.1007/s00464-025-12064-x

**Published:** 2025-08-27

**Authors:** Emily F. Simon, Christina S. Boutros, Sahra-Zahra Khan, Kamil Erozkan, Natalie Chakraborty, Kristen M. Westfall, Emily Steinhagen, Ronald Charles

**Affiliations:** Division of Colorectal Surgery, Department of Surgery, University Hospitals Cleveland Medical Center, 11100 Euclid Ave, Cleveland, OH 44106 USA

**Keywords:** Neoadjuvant chemoradiation, Total neoadjuvant therapy, Locally advanced rectal cancer, Oncologic resection, Centralized surgical care, Oncologic outcomes

## Abstract

**Background:**

Hospital operative volume has been shown to impact outcomes in complex oncologic surgeries. However, the effect of hospital volume on surgical quality and survival in patients with T4 rectal cancer following neoadjuvant chemoradiation, including total neoadjuvant therapy (TNT), remains unclear. This study evaluates the relationship between hospital volume and the achievement of optimal surgical resection (OSR) in patients with T4 rectal cancer and its subsequent impact on postoperative outcomes and survival.

**Methods:**

A retrospective cohort study was conducted using the National Cancer Database (NCDB) from 2004 to 2021. Patients with T4 rectal cancer undergoing curative-intent oncologic resection following neoadjuvant chemoradiation were included. Hospitals were categorized into low-, medium-, and high-volume tertiles based on the number of T4 rectal cancer resections performed over the study period. The primary outcome was OSR, defined as negative surgical margins and adequate lymph node yield. Multivariable logistic regression and Cox proportional hazards models were used to evaluate factors associated with OSR and survival.

**Results:**

A total of 4914 patients from 916 hospitals were analyzed. OSR rates were significantly higher in high-volume hospitals (66.0%) compared to medium- (59.3%) and low-volume hospitals (52.4%) (*p* < 0.001). On multivariable analysis, treatment at medium- (OR 1.26, 95% CI 1.09–1.45) and high-volume hospitals (OR 1.59, 95% CI 1.37–1.84) independently increased the odds of OSR. Failure to achieve OSR was associated with increased 90-day mortality (OR 2.06, 95% CI 1.16–3.64). Additionally, treatment at low- (HR 1.24, 95% CI 1.09–1.41) and medium-volume hospitals (HR 1.21, 95% CI 1.06–1.38) was associated with higher mortality risk compared to high-volume hospitals.

**Conclusion:**

Higher hospital operative volume is associated with improved surgical resection quality and survival in patients with T4 rectal cancer following neoadjuvant chemoradiation. These findings support the centralization of complex rectal cancer care to high-volume centers to optimize patient outcomes.

**Supplementary Information:**

The online version contains supplementary material available at 10.1007/s00464-025-12064-x.

The gold standard for locally advanced rectal cancer (LARC) is total neoadjuvant therapy (TNT) [1, 2]. Locally advanced rectal cancer is defined by the NCCN as MMR-stable, clinical tumor (cT) 3 or 4 disease or any cT stage with clinical nodal involvement, without distant metastasis [2]. The transition from the historical approach of surgery followed by adjuvant therapy to delivering all planned chemotherapy and radiation upfront is supported by several randomized trials investigating different TNT protocols [3–11]. These studies demonstrate improved disease-free and overall survival, reduced distant metastases, increased pathologic complete response (pCR) following surgery, and the potential for organ preservation in select cases [3–11]. Those who achieve a clinical complete response (cCR) following TNT, defined as no residual radiologic or endoscopic disease, can safely be surveyed under a non-operative management (NOM) protocol, reducing the need for morbid surgery [4, 10, 11]. However, not all LARC patients have the same potential for achieving a cCR or organ preservation. Patients with cT4 rectal cancers, for example, present unique challenges. They constitute a much smaller fraction of the patients included in the randomized trials that established TNT and organ preservation compared to cT3 patients, leaving their expected clinical course after TNT less clearly defined [5–7, 10]. Moreover, data are mixed on whether cT4 is associated with lower clinical response rates to TNT: some data show tumor stage does not impact achieving cCR, while others indicate large tumors and cT4 portend a poor response [12–15].

In patients who do not achieve a cCR or whose disease recurs after a period of surveillance, salvage surgery is indicated [10, 11]. The potential surgical advantages of TNT include decreased tumor bulk, with prior randomized control trials consistently demonstrating tumor downstaging, high rates of pCR, and comparable quality in oncologic resection parameters (mesorectal resection grade, R0 resection, circumferential resection margins) [5–7]. However, operating in a radiated field can introduce challenges. Fibrosis can obscure tissue planes, making surgery technically challenging and impacting wound healing postoperatively [16–18]. Additionally, lymph node yield, an important tenant of adequate oncologic resection, has been reported as lower following TNT [9, 19]. Furthermore, TNT with or without organ preservation is relatively new, limiting our knowledge of longer-term outcomes following salvage surgery [4]. This is particularly relevant for patients with more advanced local disease, such as cT4 tumors, who may be at greater risk for incomplete response and require complex resections, amplifying the need for data to guide best surgical practices in this challenging cohort.

In cT4 patients with residual disease following TNT, surgeons may face a dual set of operative challenges: managing bulky, locally invasive tumor and operating within a radiated field. cT4 tumors are highly heterogeneous, ranging from localized invasion of adjacent structures to extensive, fixed tumors requiring multivisceral resection. Addressing these cases demands significant operative expertise. Prior studies show that surgeons with higher case volumes achieve better outcomes in colon and rectal surgery, including improved survival rates, even after accounting for hospital volume—a factor associated with access to advanced postoperative resources and multidisciplinary expertise [20–22]. This suggests that surgical technique, including navigating obscured anatomy and challenging dissection planes, may be particularly critical for these complex cases. However, other studies have demonstrated only limited benefit from hospital volume in similar patient cohorts [23].

The current study examines patients with cT4 disease who underwent neoadjuvant chemoradiation and subsequently required oncologic resection. The hypothesis was those treated at hospitals performing higher volumes of rectal resections would experience superior surgical resections. To quantify resection quality, a composite variable, optimal surgical resection (OSR), was defined, encompassing cases with negative surgical margins and adequate lymph node yield. The primary objectives were to assess the impact of hospital operative volume on OSR rates and to identify independent associations with OSR. Secondary objectives included evaluating trends in OSR over time and determining whether operative volume and OSR were independently associated with prolonged length of stay, 30-day readmission, or mortality.

## Materials and methods

### Data source

This was a retrospective cohort study utilizing data from the National Cancer Database (NCDB). The NCDB is a clinical oncology database that captures roughly 72% of newly diagnosed cancers annually from over 1500 Commission-accredited cancer programs [24]. Data from 2004 to 2021 were analyzed.

### Patient population

Patients with tumor (T) stage 4, nodal (N) stage 0–2, and metastatic (M) stage 0 rectal adenocarcinoma were included. Patients with other tumor histology, T stage 0–3, metastatic disease, or unknown T stage were excluded. All patients were age 18 or older, and patients age 90 or older were coded as 90 years of age as predefined in the NCDB. When appropriate, patients were grouped categorically into three tertiles: those 53 or younger, 54 to 63, or 64 and older.

Other demographic variables included sex (defined as binary male/female), race (defined as non-Hispanic White, non-Hispanic Black, Hispanic and other), Charlson–Deyo Comorbidity Score (defined as an ordinal variable ranging from 0 to 3 or more, with 0 representing no comorbidities), patient residence (metropolitan, urban, rural, or unknown), insurance status (uninsured, private, Medicaid/Medicare, other government, or unknown), and income (defined as the median income of the patient’s area of residency in quartiles). The year a patient was diagnosed was defined as a categorical variable: those diagnosed from 2004 to 2009, 2010 to 2015, or 2016 to 2021.

### Treatment and surgery

Patients were further excluded if they had not undergone neoadjuvant chemoradiation or did not have definitive oncologic resection. Neoadjuvant radiation was defined using the following parameters contained within the NCDB:

- Radiation was defined as patients who were coded as having received radiation via the variable “REASON_FOR_NO_RADIATION” and “RAD_LOCATION_OF_RX.” All others were excluded. Furthermore, the dose of radiation was defined using “PHASE_I_TOTAL_DOSE,” with patients receiving less than 2000 centigray or unknown dose of radiation being excluded. Radiation course was defined as long course (4500 to 6000 centigray), short course (2000 to 3000 centigray), or other (greater than 3000 but less than 4500 or greater than 6000).
- Chemotherapy was defined as patients who underwent multi-agent chemotherapy as defined by the variable “RX_SUMM_CHEMO.” Those who did not receive chemotherapy or the type was unknown were excluded.
- Finally, variables capturing the start dates of chemotherapy, radiation, and definitive surgery were utilized to ensure all patients had received chemotherapy and radiation prior to surgery. Patients whose start date from chemotherapy or radiation to surgery was zero or negative values were excluded.

Definitive oncologic resection was defined using the variable “RX_HOSP_SURG_PRIM_SITE.” This variable captures the most definitive surgical procedure performed at the facility which submitted the patient’s record to the NCDB. This is crucial, as it ensures the location of a surgical procedures and facility location associated with a given patient align. Those who had a definitive surgery at a facility different than the reporting facility were excluded to ensure accuracy of the hospital operative volume (defined below). To further ensure one-to-one correspondence of the patients, location of surgery, and facility location, patients whose information from the NCDB arose from several facilities (captured in “PUF_MULT_SOURCE”) were also excluded. Regarding surgical procedures, patients who underwent no surgery, local destruction, local excision, or whom it was unknown if surgery was performed were excluded.

Characteristics of the facility where a patient received care were defined as facility type (community cancer program, comprehensive community cancer program, academic or research program, integrated network cancer program, or unknown). Surgical procedures included “partial proctectomy” (low anterior resection (LAR) and anterior resection (AR)), “coloanal anastomosis,” “total proctectomy” (abdominoperineal resection (APR)), “proctectomy or proctocolectomy with resection in continuity with other organs; pelvic exenteration” (pelvic exenteration), “total proctocolectomy, NOS”; “proctectomy, NOS”; and “surgery, NOS.” Surgical approach (robotic, laparoscopic, or open) was compared for patients diagnosed in 2010 or later (correlating to the introduction of the variable into the NCDB).

### Hospital volume

**Fig. 1:**
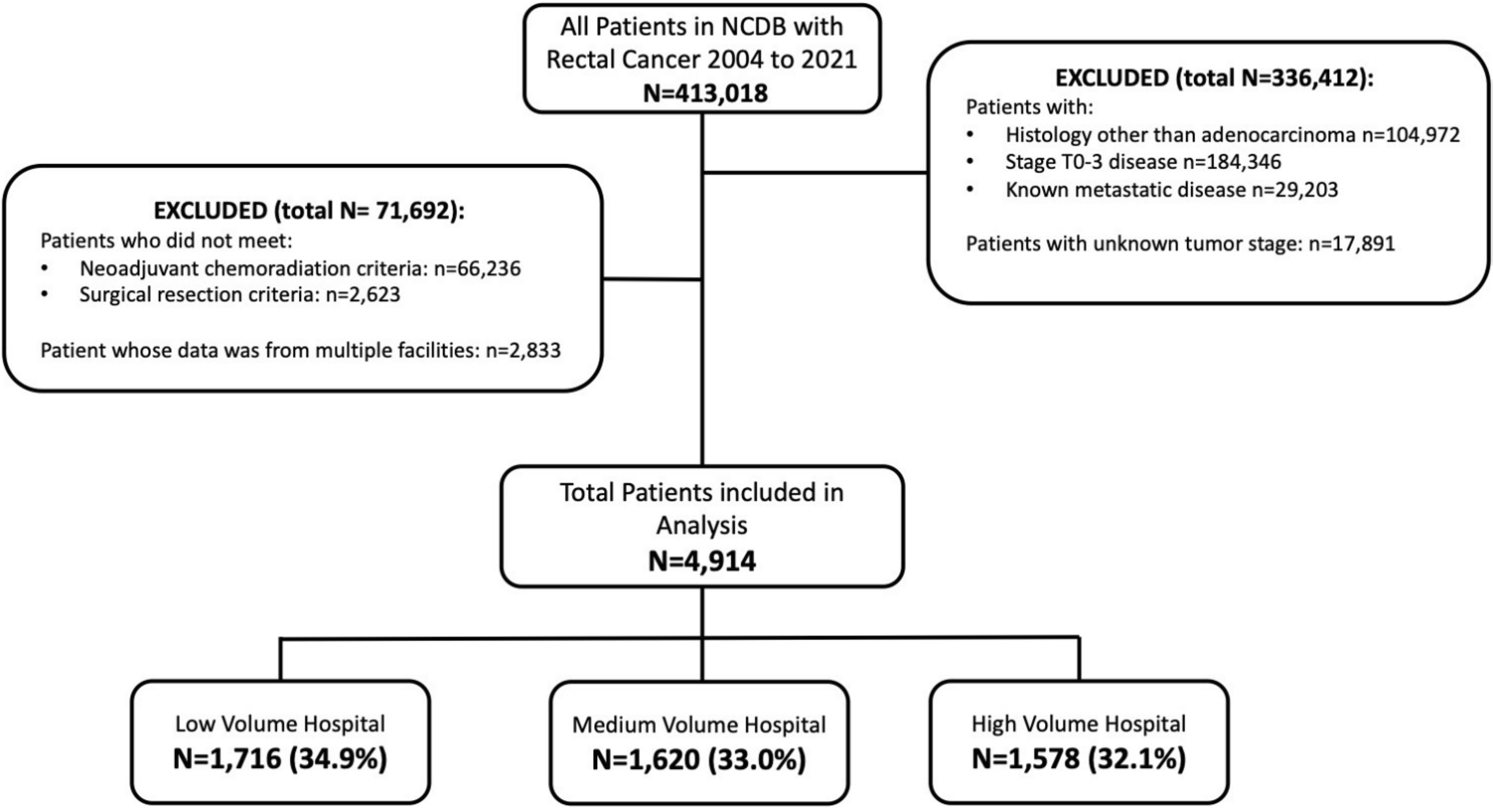
CONSORT diagram. Flowchart of patient exclusion from the national cancer database based on study criteria

Patients were grouped into tertiles based on the relative volume of surgical resection performed at the facility they received treatment. The tertiles were derived only after exclusion of all other patients had been performed, thus specifically represented the volume of T4 rectal cancer resections after neoadjuvant chemoradiation, as opposed to surgical procedures or rectal cancer operations as a whole. The volume totals represent the number of cases over the duration of the study period (i.e., 2004 to 2021). The groups were defined as low-, medium-, and high-operative hospital volume (Fig. 1).

### Outcomes

The primary outcome was a binary composite variable, labeled “optimal surgical resection” (OSR), defined as having both 12 or more lymph nodes removed (using the variable “REGIONAL_NODES_EXAMINED”) and negative surgical margins (using the variable “RX_SUMM_SURGICAL_MARGINS”) at the time of oncologic resection. If data were unknown for either variable, the patient was coded as having failed to achieve the composite outcome. Secondary outcomes included readmission within 30 days, length of hospital stay, 30-day mortality, and 90-day mortality.

### Statistical analysis

All analysis was performed using StataSE v17.0 (Statacorp LLC, College Station, TX). Univariate analysis compared demographics and surgical outcomes across the three groups of patients. Pearson’s chi-square test was used to compare categorical variables, while Kruskal–Wallis test was used for continuous variables. Multivariable logistic regression models were used to determine factors associated with achieving the composite outcome, OSR, as well as readmission in 30 days, length of stay greater than 1 week, and mortality. Hospital volume, year of diagnosis, surgical procedure (simplified as LAR/AR, APR, pelvic exenteration, or other), N stage, and selected demographic factors (Charlson–Deyo Comorbidity Score, race, sex, age) were used as independent variables. A sub-analysis was performed to explore the effect modification of hospital volume on achieving OSR by year of diagnosis by creating an interaction term between hospital volume and year of diagnosis. A Cox proportional hazards regression model was employed to analyze the relationship between surgical factors and mortality, accounting for potential confounders and time-to-event data.

## Results

### Demographics

A total of 413,018 patients diagnosed with rectal cancer between 2004 and 2021 were available in the NCDB. After exclusion, 4914 patients with T4 rectal cancer treated at 916 distinct facilities were included for the final analysis (Fig. 1). The range of case numbers at a given facility from 2004 to 2021 for T4 rectal cancer following TNT were 1 to 89. Tertiles were created using the closest whole number of procedures performed to ensure groups were as equal as possible. Low-volume hospitals performed 6 or fewer procedures, medium-volume hospitals performed between 7 and 14 procedures, and high-volume hospitals performed more than 14 procedures.

Roughly 59.7% of patients were male, 72.6% were non-Hispanic White and the median patient age was 58 years old. Most patients had low Charlson–Deyo Comorbidity (CDC) scores, with greater than 95% scoring 1 or less. Most patients had N Stage 0 or 1 (28.2% and 28.3%, respectively) relative to N Stage 2 disease (21.9%) or unknown N stage (21.7%). Patients grouped by hospital operative volume did not significantly differ regarding sex (*p* = 0.35), CDC score (*p* = 0.32), or median income (*p* = 0.9). All other demographic factors including race (*p* = 0.02), median age (*p* < 0.001), year of diagnosis (*p* < 0.001), residential location (*p* < 0.001), insurance status (*p* < 0.001), and N Stage (*p* < 0.001) differed significantly (Table 1).

**Table 1:** Univariate comparison of demographic and treatment factors across patients grouped by hospital operative volume of T4 rectal cancer resections after neoadjuvant chemoradiation

		Low volume	Medium volume	High volume	*p* value
		Total *N* = 1716 *N* (% or IQR)	Total *N* = 1620 *N* (% or IQR)	Total *N* = 1578 *N* (% or IQR)	
Sex	Male	1046 (61.0%)	966 (59.6%)	923 (58.5%)	0.035
	Female	670 (39.0%)	654 (40.4%)	655 (41.5%)	
Race	Non-Hispanic White	1238 (72.1%)	1193 (73.6%)	1136 (72.0%)	0.024
	Non-Hispanic Black	146 (8.5%)	158 (9.8%)	115 (7.3%)	
	Hispanic	158 (9.2%)	134 (8.3%)	168 (10.6%)	
	Other	174 (10.1%)	135 (8.3%)	159 (10.1%)	
Age at diagnosis		60 (52–67)	57 (50–65)	57 (49–65)	< 0.001
Year of diagnosis	2004–2009	498 (29.0%)	407 (25.1%)	328 (20.8%)	< 0.001
	2010–2015	421 (24.5%)	359 (22.2%)	353 (22.4%)	
	2016–2021	797 (46.4%)	854 (52.7%)	897 (56.8%)	
Charlson–Deyo comorbidity score	0	1335 (77.8%)	1290 (79.6%)	1275 (80.8%)	0.32
	1	310 (18.1%)	261 (16.1%)	233 (14.8%)	
	2	47 (2.7%)	43 (2.7%)	46 (2.9%)	
	3 or more	24 (1.4%)	26 (1.6%)	24 (1.5%)	
Patient residency	Metropolitan	1385 (80.7%)	1278 (78.9%)	1205 (76.4%)	< 0.001
	Urban	257 (15.0%)	248 (15.3%)	228 (14.4%)	
	Rural	32 (1.9%)	44 (2.7%)	25 (1.6%)	
	Unknown	42 (2.4%)	50 (3.1%)	120 (7.6%)	
Insurance	Uninsured	94 (5.5%)	100 (6.2%)	116 (7.4%)	< 0.001
	Private	802 (46.7%)	846 (52.2%)	855 (54.2%)	
	Medicaid or Medicare	784 (45.7%)	627 (38.7%)	564 (35.7%)	
	Other government	12 (0.7%)	26 (1.6%)	22 (1.4%)	
	Unknown	24 (1.4%)	21 (1.3%)	21 (1.3%)	
Income	< $46,277	274 (16.0%)	272 (16.8%)	257 (16.3%)	0.09
	$46,277-$57,856	345 (20.1%)	295 (18.2%)	294 (18.6%)	
	$57,857-$74,062	361 (21.0%)	386 (23.8%)	341 (21.6%)	
	$74,063 +	481 (28.0%)	464 (28.6%)	493 (31.2%)	
	Unknown	255 (14.9%)	203 (12.5%)	193 (12.2%)	
Nodal stage	N Stage 0	548 (32.1%)	449 (27.8%)	385 (24.4%)	< 0.001
	N Stage 1	459 (26.9%)	447 (27.6%)	479 (30.4%)	
	N Stage 2	281 (16.4%)	360 (22.2%)	432 (27.4%)	
	N Stage unknown	421 (24.6%)	362 (22.4%)	279 (17.7%)	
Facility type	Community cancer program	192 (11.2%)	6 (0.4%)	0 (0.0%)	< 0.001
	Comprehensive community cancer program	779 (45.4%)	584 (36.0%)	295 (18.7%)	
	Academic/research program	260 (15.2%)	693 (42.8%)	981 (62.2%)	
	Integrated network cancer program	405 (23.6%)	234 (14.4%)	182 (11.5%)	
	Unknown	80 (4.7%)	103 (6.4%)	120 (7.6%)	
Radiation course	Long course (4500–6000 centigray)	1466 (85.4%)	1296 (80.0%)	1215 (77.0%)	< 0.001
	Short course (2000–3000 centigray)	46 (2.7%)	72 (4.4%)	89 (5.6%)	
	Other	204 (11.9%)	252 (15.6%)	274 (17.4%)	
Surgical procedure	Low anterior resection/anterior resection	1001 (58.3%)	884 (54.6%)	797 (50.5%)	< 0.001
	Coloanal anastomosis	86 (5.0%)	102 (6.3%)	101 (6.4%)	
	Abdominoperineal resection	461 (26.9%)	409 (25.2%)	425 (26.9%)	
	Total proctocolectomy, NOS	34 (2.0%)	33 (2.0%)	39 (2.5%)	
	Pelvic exenteration	90 (5.2%)	160 (9.9%)	191 (12.1%)	
	Proctectomy, NOS	10 (0.6%)	9 (0.6%)	8 (0.5%)	
	Surgery, NOS	34 (2.0%)	23 (1.4%)	17 (1.1%)	
Surgical approach*	Open	542 (44.5%)	547 (45.1%)	578 (46.2%)	0.28
	Laparoscopic	292 (24.0%)	263 (21.7%)	254 (20.3%)	
	Robotic	384 (31.5%)	403 (33.2%)	418 (33.4%)	
Lymph node yield	< 12	661 (38.5%)	515 (31.8%)	413 (26.2%)	< 0.001
	12 or more	1038 (60.5%)	1095 (67.6%)	1153 (73.1%)	
	Unknown	17 (1.0%)	10 (0.6%)	12 (0.8%)	
Surgical margins	Negative	1488 (86.7%)	1411 (87.1%)	1408 (89.2%)	0.18
	Positive	206 (12.0%)	193 (11.9%)	157 (9.9%)	
	Unknown	22 (1.3%)	16 (1.0%)	13 (0.8%)	
Optimal surgical resection	Received	900 (52.4%)	961 (59.3%)	1042 (66.0%)	< 0.001
	Failed	816 (47.6%)	659 (40.7%)	536 (34.0%)	

Categorical variables compared using chi−square; number of patients and percentage of total patients reported per category. Continuous variables compared using Kruskal–Wallis; median and interquartile range reported. *Patients diagnosed 2010 or later

### Treatment and surgical factors

Patients in the low-operative volume hospital group received care most often at Comprehensive Community Cancer Programs (45.4%), compared to Academic or Research Centers for medium- (42.8%) and high-operative volume hospitals (62.25; *p* < 0.001) (Table 1).

The groups differed significantly regarding radiation course (*p* < 0.001), but greater than 75% of patients across all groups received long-course radiation. Similarly, though statistically different across the three groups (*p* < 0.001), roughly half of patients underwent a LAR or AR, followed by roughly a quarter undergoing APRs. Of patients diagnosed in 2010 or later, the groups did not differ regarding surgical approach, with most undergoing open surgery (*p* = 0.28) (Table 1).

Patients in the low-operative volume hospital group had the lowest rate of adequate lymph node yield at 60.5%, compared to 67.6% and 73.1% in medium- and high-operative volume groups, respectively (*p* < 0.001). The groups did not differ significantly regarding negative surgical margins, with all groups achieve negative margins in over 86% of cases (*p* = 0.18) (Table 1).

### Primary outcomes

The groups differed significantly regarding the composite outcome of OSR, with the low-operative volume hospital group receiving OSR in 52.4% of cases, compared to 59.3% and 66.0% in medium and high-operative volume groups, respectively (*p* < 0.001) (Table 1).

On multivariable logistic regression modeling, both medium- and high-operative volume groups had increased odds of receiving OSR (OR 1.26, CI 1.09–1.45; OR 1.59, CI 1.37–1.84, respectively) compared to the low-operative volume group. Later year of diagnosis (2016–2021: OR 2.90, CI 2.41–3.48) and higher nodal stage (stage 1: OR 1.20, CI 1.02–1.40; stage 2: OR 1.28, CI 1.07–1.54) also increased odds of achieving OSR. APR and pelvic exenteration, as compared to LAR or AR, decreased the odds of an OSR (OR 0.79, CI 0.69–0.90; OR 0.76, CI 0.62–0.95, respectively). Older age (64 or more: OR 0.71, CI 0.61–0.82) and non-Hispanic Black race (OR 0.74, CI 0.60–0.92) also decreased odds of receiving an OSR. Radiation course and CDC score were not associated with receiving OSR (Table 2).

**Table 2:** Multivariable logistic regression model predicting receiving optimal surgical resection following oncologic rectal surgery

		Odds ratio	*p* value	Confidence interval	
Hospital operative volume	Low	Reference			
	Medium	1.26	0.00	1.09	− 1.45
	High	1.59	0.00	1.37	− 1.84
Year of diagnosis	2004–2009	Reference			
	2010–2015	2.14	0.00	1.77	− 2.57
	2016–2021	2.90	0.00	2.41	− 3.48
Age	< 54	Reference			
	54 to 63	0.74	0.00	0.64	− 0.86
	64 or older	0.71	0.00	0.61	− 0.82
Surgical procedure	LAR/AR	Reference			
	APR	0.79	0.00	0.69	− 0.90
	Pelvic exenteration	0.76	0.01	0.62	− 0.95
	Other	0.91	0.65	0.60	− 1.37
Race	Non-Hispanic White	Reference			
	Non-Hispanic Black	0.74	0.01	0.60	− 0.92
	Hispanic	0.97	0.77	0.79	− 1.20
	Other	1.06	0.60	0.86	− 1.30
Radiation course	Long course	Reference			
	Short course	1.23	0.20	0.90	− 1.69
	Other	1.01	0.92	0.85	− 1.19
Nodal stage	N Stage 0	Reference			
	N Stage 1	1.20	0.03	1.02	− 1.40
	N Stage 2	1.28	0.01	1.07	− 1.54
	N Stage unknown	1.14	0.17	0.95	− 1.38
Charlson–Deyo comorbidity score	0	Reference			
	1	0.93	0.35	0.79	− 1.09
	2	0.81	0.24	0.56	− 1.15
	3 or more	0.92	0.75	0.57	− 1.50

*LAR* low anterior resection, *AR* anterior resection, *APR* abdominoperineal resection

On secondary analysis of the model with an interaction term between hospital operative volume and year of diagnosis, patients diagnosed between 2016 and 2021 remained with the highest odds of receiving an OSR, with a graded increase in odds from low- to high-operative volume groups relative to the reference category of low-operative volume from 2004 to 2009 (2016 to 2021 low: OR 3.27, CI 2.52–4.24; medium: OR 3.70, CI 2.85–4.81; high: OR 4.42, CI 3.38–5.76) (Table 3).

**Table 3:** Multivariable logistic regression model predicting receiving optimal surgical resection following oncologic rectal surgery with interaction effect between hospital volume and year of diagnosis

			Odds ratio	*p* value	Confidence interval	
Hospital operative volume by year of diagnosis	Low	2004–2009	Reference			
		2010–2015	1.97	0.00	1.49	− 2.61
		2016–2021	3.27	0.00	2.52	− 4.24
	Medium	2004–2009	1.34	0.04	1.02	− 1.76
		2010–2015	2.79	0.00	2.08	− 3.75
		2016–2021	3.70	0.00	2.85	− 4.81
	High	2004–2009	1.67	0.00	1.25	− 2.22
		2010–2015	4.07	0.00	3.00	− 5.53
		2016–2021	4.42	0.00	3.38	− 5.76
Age		< 54	Reference			
		54 to 63	0.74	0.00	0.64	− 0.86
		64 or older	0.71	0.00	0.61	− 0.82
Surgical procedure		LAR/AR	Reference			
		APR	0.79	0.00	0.69	− 0.90
		Pelvic exenteration	0.76	0.01	0.61	− 0.94
		Other	0.90	0.63	0.59	− 1.37
Race		Non-Hispanic White	Reference			
		Non-Hispanic Black	0.74	0.01	0.60	− 0.92
		Hispanic	0.97	0.80	0.79	− 1.20
		Other	1.06	0.58	0.86	− 1.30
Radiation course		Long course	Reference			
		Short course	1.25	0.17	0.91	− 1.71
		Other	1.01	0.92	0.85	− 1.19
Nodal stage		N Stage 0	Reference			
		N Stage 1	1.19	0.03	1.02	− 1.40
		N Stage 2	1.29	0.01	1.07	− 1.55
		N Stage unknown	1.14	0.17	0.94	− 1.38
Charlson–Deyo comorbidity score		0	Reference			
		1	0.93	0.35	0.79	− 1.09
		2	0.80	0.22	0.56	− 1.15
		3 or more	0.93	0.76	0.57	− 1.51

*LAR* low anterior resection, *AR* anterior resection, *APR* abdominoperineal resection

### Secondary outcomes

The groups differed significantly in unplanned readmissions (*p* < 0.001) and length of stay greater than 1 week (*p* < 0.01). However, the absolute differences were small across all three groups and partially driven by a significant minority of patients with unknown data. Namely, 6.1% of patients in both low- and high-operative volume groups had unplanned readmissions compared to 6.9% in the medium-operative volume group while 26.7%, 27.4%, and 26.6% of patients in low-, medium- and high-operative volume groups, respectively, stayed longer than 1 week. The groups did not differ in terms of 30- or 90-day mortality (*p* = 0.97, *p* = 0.46; respectively).

On multivariable logistic regression modeling, failure to receive OSR increased a patient’s odds of having a length of stay greater than 1 week (OR 1.27, CI 1.11–1.46). Other variables that increased odds of long length of stay included older age (64 or older: OR 1.54, CI 1.30–1.82), undergoing an APR (OR 1.35, CI 1.16–1.57) or pelvic exenteration (OR 3.03, CI 2.42–3.79), non-Hispanic Black race (OR 1.54, CI 1.21–1.94), and CDC score of 1 (OR 1.25, CI 1.05–1.49). Year of diagnosis between 2016 and 2021 decreased the odds of long length of stay (OR 0.75, CI 0.61–0.92).

OSR was not associated with 30-day unplanned readmission. Non-Hispanic Black race (OR 1.60, CI 1.12–2.30) and CDC score of 1 (OR 1.75, CI 1.33–2.31) increased odds of readmission.

Hospital operative volume and OSR were not associated with 30-day mortality on multivariable logistic regression modeling. Hospital operative volume remained non-correlative of 90-day mortality; however, patients who failed to receive OSR had double the odds of 90-day mortality compared to those who received OSR (OR 2.06, CI 1.16–3.64). On Cox proportional hazards regression modeling, both failure to receive OSR (HR 1.43, CI 1.28–1.59) and low- or medium-operative volume groups (HR 1.24, CI 1.09–1.41; HR 1.21, CI 1.06–1.38, respectively) had increased risk of mortality. Older age (64 or older: HR 1.57, CI 1.37–1.78), undergoing an APR (HR 1.20, CI 1.07–1.35) or pelvic exenteration (HR 1.39, CI 1.15–1.67) and higher CDC score also increased mortality risk (score 3 or more: HR 1.87, CI 1.29–2.73) (Table 4).

**Table 4:** Cox proportional hazards regression model predicting mortality after oncologic rectal surgery

		Hazard ratio	*p* value	Confidence interval	
Optimal surgical resection	Achieved	Reference			
	Failed	1.43	0.00	1.28	− 1.59
Hospital operative volume	Low	1.24	0.00	1.09	− 1.41
	Medium	1.21	0.01	1.06	− 1.38
	High	Reference			
Year of diagnosis	2004–2009	Reference			
	2010–2015	0.94	0.39	0.82	− 1.08
	2016–2021	0.87	0.12	0.74	− 1.03
Age	< 54	Reference			
	54 to 63	1.27	0.00	1.11	− 1.45
	64 or older	1.57	0.00	1.37	− 1.78
Surgical procedure	LAR/AR	Reference			
	APR	1.20	0.00	1.07	− 1.35
	Pelvic exenteration	1.39	0.00	1.15	− 1.67
	Other	1.12	0.45	0.83	− 1.52
Race	Non-Hispanic White	Reference			
	Non-Hispanic Black	1.07	0.45	0.90	− 1.28
	Hispanic	0.97	0.78	0.79	− 1.19
	Other	0.90	0.22	0.75	− 1.07
Radiation course	Long Course	Reference			
	Short Course	1.44	0.05	1.00	− 2.07
	Other	1.12	0.12	0.97	− 1.28
Nodal stage	N Stage 0	Reference			
	N Stage 1	1.07	0.37	0.92	− 1.24
	N Stage 2	1.02	0.87	0.84	− 1.23
	N Stage Unknown	1.24	0.00	1.09	− 1.43
Charlson–Deyo comorbidity score	0	Reference			
	1	1.14	0.06	1.00	− 1.29
	2	1.56	0.00	1.18	− 2.07
	3 or more	1.87	0.00	1.29	− 2.73

*LAR* low anterior resection, *AR* anterior resection, *APR* abdominoperineal resection

## Discussion

The current study demonstrates that higher hospital operative volume of T4 rectal cancer following neoadjuvant chemoradiation is significantly associated with improved rates of OSR. Operative volume was also an incremental, independent correlate of achieving OSR, with both medium-operative volume (OR 1.26, CI 1.09 –1.45) and high-operative volume centers (OR 1.59, CI 1.37–1.84) portending increased odds of achieving OSR compared to low-operative volume hospitals. The association between hospital operative volume and OSR remained significant over time, with patients diagnosed more recently and at high-operative volume centers experiencing the greatest improvements (2016–2021 high: OR 4.42, CI 3.38–5.76). Finally, although hospital operative volume was not associated with 30- or 90-day mortality on multivariable logistic regression modeling, it was associated with a small but significant reduction in the risk of mortality on Cox proportional hazards regression. Both low- (HR 1.24, CI 1.09–1.41) and medium-operative volume hospitals (HR 1.21, CI 1.06–1.38) demonstrated increased mortality risk compared to high-operative volume hospitals. OSR itself was a strong correlate of survival, with failure to achieve OSR associated with increased odds of 90-day mortality (OR 2.06, CI 1.16–3.64) and a higher hazard of death on Cox regression (HR 1.43, CI 1.28–1.59). These results support the concept that surgical quality directly impacts oncologic outcomes and taken together, provides evidence of a volume-outcome relationship following resection of T4 rectal cancer after neoadjuvant chemoradiation.

The relationship between hospital or surgeon volume and rectal cancer outcomes has been previously investigated, with variable results depending on the population and context. In a US study of patients with LARC undergoing postoperative chemoradiation, hospital volume did not independently correlate with recurrence or survival for patients who completed appropriate adjuvant therapy [25]. However, among those who did not complete adjuvant therapy, treatment at low-volume centers was associated with higher recurrence rates and a trend toward increased mortality [25]. In a post-hoc analysis of a German randomized trial comparing pre- versus postoperative chemoradiotherapy for LARC, hospital operative volume was associated with improved 10-year overall survival, while individual surgeon volume correlated with better local tumor control [26]. Two large European cancer registry studies also demonstrated superior survival outcomes [21, 22]. The first showed improved patient survival at 90 days, 1 year, and 5 years after resection for patients with stage I–III rectal cancer treated at high-volume hospitals [26]. The second focused on all patients undergoing proctectomy over the course of 5 years, reporting an inverse linear relationship between low postoperative mortality and high-volume centers [22]. This study is particularly relevant to ours as it focused on surgery and surgical volume as the independent variable. The variability in findings across studies likely reflects differences in study populations, including wide variation in tumor stage and perioperative treatment. Our study adds to this body of literature by focusing on a narrowly defined and uniquely complex population—patients with cT4 rectal cancer treated with neoadjuvant chemoradiation—offering important insight into how volume influences outcomes in this high-risk cohort.

Given the evidence supporting improved outcomes at high-volume centers, it is important to ensure equitable access to these centers for all patients. Both in the USA and internationally, initiatives have been introduced to promote the centralization of complex oncologic surgery or to define case minimums for specific procedures [27–30]. In Europe, for example, several countries have adopted formal centralization of pancreatic surgery, a shift associated with a roughly 6% decrease in postoperative mortality over a decade in the Netherlands [28, 31]. In the USA, the Leapfrog Group has published annual minimum volume recommendations for many oncologic surgeries, including rectal cancer, since the early 2000s [27, 29, 30]. More recently, the National Accreditation Program for Rectal Cancer (NAPRC) was established to set hospital standards and improve rectal cancer care quality [32]. Compliance with NAPRC standards is estimated to save up to 300 lives annually, with a potential 10% reduction in surgical mortality at accredited hospitals [32–34]. While centralization offers numerous potential benefits, concerns about the burden placed on patients, especially those living far from specialized centers, must not be overlooked. One study estimated that centralization would increase the mean distance of travel for rectal cancer patients by 9.5 miles, with a greater than 300% increase in the number of patients traveling over 50 miles for surgery [35]. Thus, implementation should be carefully balanced against competing considerations. As these efforts evolve, they are likely to have the greatest impact on patients requiring the most complex care—such as those with cT4 rectal cancer—where surgical expertise and multidisciplinary collaboration are essential for optimizing outcomes.

This study is limited by its retrospective design and reliance on a large national database, the NCDB. Due to the inherent granularity of NCDB data, a patient’s treatment course and surgical outcomes were defined using the best available surrogate variables. Due to inherent limitations of the NCDB and the inclusion of a study period that largely predated the routine adoption of TNT, the study population was broadly defined as patients who received neoadjuvant chemoradiation, rather than those treated with the contemporary TNT protocols aimed at organ preservation. Importantly, the core surgical challenges that motivated this study’s hypothesis—operating in a radiated field with or without residual tumor—apply regardless of whether every patient strictly met modern TNT criteria. Another limitation is the inability to account for surgeon-specific outcomes. While hospital operative volume serves as a reasonable proxy for surgeon volume, other factors such as institutional expertise, nursing care, multidisciplinary coordination, and standardized postoperative protocols may also influence outcomes. This is further supported by the correlation between high-volume centers and academic or research facilities, highlighting the broader context of institutional experience. Additionally, it is important to note that high-volume centers performed more than double the proportion of pelvic exenterations compared to low-volume centers (12.1% vs. 5.2%, respectively), reflecting their role in managing the most complex cases. Although our models adjust for surgical procedure type, the favorable impact of receiving surgery at a high-volume hospital may still be underestimated given the unique complexity and risks associated with these procedures.

Hospital operative volume is strongly associated with achieving optimal surgical resection (OSR) in patients with cT4 rectal cancer who undergo oncologic resection following neoadjuvant chemoradiation, even in the modern era. This suggests that despite the tremendous oncologic success of TNT programs, high-quality surgery (negative margins and adequate node harvesting) still matters. Both the current study and prior literature support that achieving OSR significantly impacts patient outcomes, including survival. Encouragingly, OSR rates have improved over time; however, the advantages of experience and institutional expertise at high-volume hospitals have not been fully offset by these temporal improvements. These findings reinforce the critical importance of surgical experience and lend support to the consideration of regionalizing surgical care to high-volume centers for patients with T4 rectal cancer who require oncologic resection following neoadjuvant treatment, including TNT. Future research, including surgeon-specific analyses, will be essential to further clarify the interplay between surgeon case volume, institutional resources, and surgical quality in this complex patient population.

## Supplementary Information

Below is the link to the electronic supplementary material.

Supplementary file1 (DOCX 27 kb)
