# Supplementary material for: Hospital operative volume impacts surgical outcomes for patients with T4 rectal cancer following neoadjuvant chemoradiation: a national cancer database analysis
Source: Surg Endosc. 2025 Aug 27;39(10):6903–14. doi: 10.1007/s00464-025-12064-x (PMC12500830; doi:10.1007/s00464-025-12064-x)
Supplement: Supplementary file 1 — Supplementary file1 (DOCX 27 kb) [file 464_2025_12064_MOESM1_ESM.docx]

**Supplemental Materials:**

Table: Univariate comparison of postoperative factors across patients grouped by hospital operative volume of T4 rectal cancer resections after TNT

| Column 1  Column 2 | | **Low Volume** | **Medium Volume** | **High Volume** | ***p-value*** |
| --- | --- | --- | --- | --- | --- |
|  |  | Total N=1,716  N (%) | Total N=1,620  N (%) | Total N=1,578  N (%) |  |
| **Unplanned Readmission** | No Unplanned Readmission | 1,580 (92.1%) | 1,489 (91.9%) | 1,478 (93.7%) | *<0.001* |
|  | Unplanned Readmission | 104 (6.1%) | 111 (6.9%) | 96 (6.1%) |  |
|  | Unknown | 32 (1.9%) | 20 (1.2%) | 4 (0.3%) |  |
| **Length of Stay** | 1 week or less | 1,032 (60.1%) | 997 (61.5%) | 1,020 (64.6%) | *<0.01* |
|  | >1 week | 458 (26.7%) | 444 (27.4%) | 419 (26.6%) |  |
|  | Unknown | 226 (13.2%) | 179 (11.0%) | 139 (8.8%) |  |
| **30-Day Mortality** | Alive | 1,504 (88.3%) | 1,437 (89.0%) | 1,394 (88.5%) | *0.97* |
|  | Dead | 7 (0.4%) | 7 (0.4%) | 6 (0.4%) |  |
|  | Unknown | 193 (11.3%) | 171 (10.6%) | 175 (11.1%) |  |
| **90-Day Mortality** | Alive | 1,481 (87.2%) | 1,417 (88.1%) | 1,370 (88.0%) | *0.46* |
|  | Dead | 24 (1.4%) | 20 (1.2%) | 12 (0.8%) |  |
|  | Unknown | 193 (11.4%) | 171 (10.6%) | 175 (11.2%) |  |

Table: Multivariable Logistic Regression Model predicting length of stay greater than 1 week following oncologic rectal surgery

|  | | **Odds Ratio** | **p-value** | **Confidence interval** | |
| --- | --- | --- | --- | --- | --- |
| **Optimal Surgical Resection** | Received | reference |  |  |  |
|  | Failed | 1.27 | 0.00 | 1.11 | - 1.46 |
| **Hospital Operative Volume** | Low | reference |  |  |  |
|  | Medium | 1.03 | 0.71 | 0.88 | - 1.21 |
|  | High | 0.96 | 0.62 | 0.81 | - 1.13 |
| **Year of Diagnosis** | 2004-2009 | reference |  |  |  |
|  | 2010-2015 | 1.02 | 0.81 | 0.84 | - 1.25 |
|  | 2016-2021 | 0.75 | 0.01 | 0.61 | - 0.92 |
| **Age** | <54 | reference |  |  |  |
|  | 54 to 63 | 1.10 | 0.25 | 0.93 | - 1.31 |
|  | 64 or older | 1.54 | 0.00 | 1.30 | - 1.82 |
| **Surgical Procedure** | LAR/AR | reference |  |  |  |
|  | APR | 1.35 | 0.00 | 1.16 | - 1.57 |
|  | Pelvic exenteration | 3.03 | 0.00 | 2.42 | - 3.79 |
|  | Other | 0.97 | 0.89 | 0.60 | - 1.56 |
| **Race** | Non-Hispanic White | reference |  |  |  |
|  | Non-Hispanic Black | 1.54 | 0.00 | 1.21 | - 1.94 |
|  | Hispanic | 1.23 | 0.08 | 0.97 | - 1.56 |
|  | Other | 1.16 | 0.19 | 0.93 | - 1.46 |
| **Radiation Course** | Long Course | reference |  |  |  |
|  | Short Course | 1.05 | 0.80 | 0.74 | - 1.48 |
|  | Other | 1.10 | 0.32 | 0.91 | - 1.32 |
| **Nodal Stage** | N Stage 0 | reference |  |  |  |
|  | N Stage 1 | 0.91 | 0.29 | 0.76 | - 1.09 |
|  | N Stage 2 | 0.84 | 0.09 | 0.68 | - 1.03 |
|  | N Stage Unknown | 0.99 | 0.91 | 0.81 | - 1.21 |
| **Charlson-Deyo Comorbidity Score** | 0 | reference |  |  |  |
|  | 1 | 1.25 | 0.01 | 1.05 | - 1.49 |
|  | 2 | 0.92 | 0.68 | 0.61 | - 1.38 |
|  | 3 or more | 1.25 | 0.41 | 0.74 | - 2.13 |

^LAR=low anterior resection, AR=anterior resection, APR=abdominoperineal resection^

Table: Multivariable Logistic Regression Model predicting unplanned 30-Day readmission following oncologic rectal surgery

|  | | **Odds Ratio** | **p-value** | **Confidence interval** | |
| --- | --- | --- | --- | --- | --- |
| **Optimal Surgical Resection** | Achieved | reference |  |  |  |
|  | Failed | 0.99 | 0.94 | 0.78 | - 1.26 |
| **Hospital Operative Volume** | Low | reference |  |  |  |
|  | Medium | 1.14 | 0.35 | 0.86 | - 1.51 |
|  | High | 1.00 | 0.99 | 0.75 | - 1.35 |
| **Year of Diagnosis** | 2004-2009 | reference |  |  |  |
|  | 2010-2015 | 1.08 | 0.68 | 0.75 | - 1.57 |
|  | 2016-2021 | 0.98 | 0.91 | 0.68 | - 1.41 |
| **Age** | <54 | reference |  |  |  |
|  | 54 to 63 | 0.98 | 0.91 | 0.73 | - 1.32 |
|  | 64 or older | 1.23 | 0.16 | 0.92 | - 1.65 |
| **Surgical Procedure** | LAR/AR | reference |  |  |  |
|  | APR | 0.97 | 0.84 | 0.74 | - 1.27 |
|  | Pelvic exenteration | 1.30 | 0.17 | 0.90 | - 1.90 |
|  | Other | 0.61 | 0.34 | 0.22 | - 1.68 |
| **Race** | Non-Hispanic White | reference |  |  |  |
|  | Non-Hispanic Black | 1.60 | 0.01 | 1.12 | - 2.30 |
|  | Hispanic | 1.20 | 0.37 | 0.81 | - 1.77 |
|  | Other | 0.66 | 0.10 | 0.41 | - 1.08 |
| **Radiation Course** | Long Course | reference |  |  |  |
|  | Short Course | 1.11 | 0.70 | 0.64 | - 1.94 |
|  | Other | 1.03 | 0.84 | 0.74 | - 1.44 |
| **Nodal Stage** | N Stage 0 | reference |  |  |  |
|  | N Stage 1 | 0.98 | 0.88 | 0.73 | - 1.31 |
|  | N Stage 2 | 0.76 | 0.12 | 0.53 | - 1.08 |
|  | N Stage Unknown | 0.61 | 0.01 | 0.41 | - 0.90 |
| **Charlson-Deyo Comorbidity Score** | 0 | reference |  |  |  |
|  | 1 | 1.75 | 0.00 | 1.33 | - 2.31 |
|  | 2 | 1.77 | 0.06 | 0.97 | - 3.21 |
|  | 3 or more | 1.17 | 0.74 | 0.46 | - 2.96 |

^LAR=low anterior resection, AR=anterior resection, APR=abdominoperineal resection^
